# IDENTIFYING PREDICTORS OF RESILIENCE IN SINGLE-ARM STUDIES OF PRE–POST CHANGE

**DOI:** 10.1093/geroni/igad104.1823

**Published:** 2023-12-21

**Authors:** Ravi Varadhan, Jiafeng Zhu, Karen Bandeen-Roche

**Affiliations:** Johns Hopkins University School of Medicine, Baltimore, Maryland, United States; Northwestern University, Chicago, Illinois, United States; Johns Hopkins University, Baltimore, Maryland, United States

## Abstract

With advancing age, older adults are at a greater risk of experiencing significant stressors. While frailty is the increased vulnerability to stressors, resilience is the ability to respond well to a major stressor. An important goal of geriatric research is to identify factors which influence resilience to stressors. Studies of resilience in older adults are typically conducted using a single-arm design where everyone experiences the stressor. In such designs, resilience is typically quantified as the degree of recovery in physical/cognitive/psychological functions after the stressor. The simplistic approach of regressing change versus baseline yields biased estimates due to mathematical coupling and regression-to-the-mean. Correction is necessary for eliminating the bias and drawing valid inferences regarding the effect of covariates and baseline status on pre-post change. We present a simple method to correct this bias. We extend the method to include covariates. Our approach considers a counterfactual control group and involves sensitivity analyses to evaluate different settings of control group parameters. We illustrate the method using a large, registry of older adults (N=7,239) who underwent total knee replacement (TKR). We demonstrate how external data can be utilized to constrain the sensitivity analysis. Our analysis indicates that baseline (pre-stressor) function was not strongly linked to recovery after TKR. Among the covariates, only age had a consistent effect on post-stressor recovery. A main takeaway of our work is that studies of resilience should consider, either directly or indirectly, the use of an appropriate control group.

